# Adiponectin in relation to childhood myeloblastic leukaemia

**DOI:** 10.1038/sj.bjc.6602896

**Published:** 2006-01-10

**Authors:** E Petridou, C S Mantzoros, N Dessypris, S K Dikalioti, D Trichopoulos, the Childhood Hematology Oncology Group

**Affiliations:** 1 Department of Hygiene and Epidemiology, Athens University Medical School, 75 Mikras Asias Str, Goudi, Athens 11527, Greece; 2 Department of Epidemiology, Harvard School of Public Health, Boston, MA 02115, USA; 3 Division of Endocrinology, Diabetes and Metabolism, Department of Internal Medicine, Beth Israel Deaconess Medical Center, Harvard Medical School, Boston, MA, USA

**Keywords:** adiponectin, childhood, leukaemia

## Abstract

Adiponectin, an adipocyte-specific secretory protein known to induce apoptosis, has been reported to be inversely related to breast and endometrial cancers and recently found to inhibit proliferation of myeloid but not lymphoid cell lines. We hypothesised that adiponectin may be inversely associated with acute myeloblastic leukaemia (AML), but not with acute lymphoblastic leukaemia of B (ALL-B) or T (ALL-T) cell origin in children. Blood samples and clinical information were collected over the period 1996–2000 from 201 children (0–14 years old) with leukaemia (22 AML, 161 ALL-B and 18 ALL-T cases) through a national network of childhood Hematology-Oncology units in Greece and from 201 controls hospitalised for minor pediatric ailments. Serum adiponectin levels were measured under code, at the Beth Israel Deaconess Medical Center, Boston, MA, USA using a radioimmunoassay procedure. Each of the three leukaemia groups was compared with the control group through multiple logistic regression. Odds ratios (OR) and 95% confidence intervals (95% CI) for an increase of adiponectin equal to 1 s.d. among controls were estimated controlling for gender, age, as well as for height and weight, expressed in age–gender-specific centiles of Greek growth curves. Adiponectin was inversely associated with AML (OR=0.56; 95% CI, 0.34–0.94), whereas it was not significantly associated with either ALL-B (OR=0.88; 95% CI, 0.71–1.10) or ALL-T (OR=1.08; 95% CI, 0.67–1.72). Biological plausibility and empirical evidence point to the importance of this hormone in the pathogenesis of childhood AML.

With the exception of ionizing radiation and genetic abnormalities, the aetiology of childhood leukaemia has not yet been fully elucidated. It also remains unknown whether the principal forms of childhood leukaemia, namely acute lymphoblastic leukaemia of B-cell origin (ALL-B), acute lymphoblastic leukaemia of T-cell origin (ALL-T) and acute myeloblastic leukaemia (AML) have similar aetiologies and/or pathogenesis. Most aetiologic research on childhood leukaemia has focused on ALL due to its much higher frequency in comparison to that of AML (Petridou and Trichopoulos, 2002; McNally and Eden, 2004; Randolph, 2004).

Adiponectin is an adipocyte-secreted hormone which has been found to induce apoptosis and suppress angiogenesis (Cao *et al*, 1999; Brakenhielm *et al*, 2004). This hormone has also been inversely associated with both adult forms of cancer that have been epidemiologically investigated, namely breast cancer (Miyoshi *et al*, 2003; Mantzoros *et al*, 2004b) and endometrial cancer (Petridou *et al*, 2003; Dal Maso *et al*, 2004). In a recent *in vitro* study Yokota *et al* have investigated the functions of adiponectin in haematopoiesis and found that adiponectin predominantly inhibits proliferation of myeloid cell lines, and induces apoptosis in myelomonocytic leukaemia lines, but did not suppress proliferation of erythroid or lymphoid cell lines. We have thus hypothesised that adiponectin might be inversely associated with AML, but not with ALL. We have evaluated this hypothesis by studying 201 children with incident leukaemia and 201 appropriate control children enrolled by a nation-wide network of Pediatric Hematology-Oncology Departments in Greece.

## MATERIALS AND METHODS

A national network comprising all six Childhood Hematology-Oncology Departments operating in Greece has been established and has coordinated epidemiological research for the last 15 years (Petridou *et al*, 1993, 1996). In the present study, we have included childhood leukaemia cases (0–14 years old) first diagnosed in Greece during a 5-year period (1996–2000). Children from one of the two Departments in Thessaloniki, however, were not included for administrative reasons. Thus, the study was based on 281 cases of childhood leukaemia diagnosed in five Departments, three in Athens, one in Thessaloniki and one in Crete. Of these cases, 80 were not included, because informed consent could not be obtained before the onset of therapy or laboratory samples were inappropriate or inadequate. These exclusions are unlikely to have introduced selection bias because it was based on technical and administrative reasons. Moreover, serum ADP levels could not impact on patients' decisions to participate in our study; thus it is not possible to have any influence on the reported results. Of the remaining 201 cases of childhood leukaemia, 161 were of B-cell origin (ALL-B), 18 of T-cell origin (ALL-T) and 22 had AML. We have not included in the present study cases with Down's syndrome, Fanconi anaemia, other predisposing to leukaemia genetic conditions and children with leukaemia as a second cancer or cases with a previous history of myelodysplasia.

For each child with leukaemia, a control child of the same gender and similar age (±6 months) was sought among those concurrently admitted to the Pediatric Departments of the same Hospitals for minor pediatric ailments. For only 11 of them the parents were unable or unwilling to provide informed consent and these were properly substituted. Among the 201 controls, 88 were admitted for mild respiratory conditions, viral infections or allergy (bronchitis, asthma, urticaria), 35 for gastrointestinal or genitourinary conditions (gastroenteritis, abdominal pain, urinary tract infections), 31 for nervous system conditions (febrile seizures, transient loss of consciousness, dizziness, headache), 21 for injuries including acute poisoning and near drowning, six for muscle-osteoarticular conditions (arthritis, joint pain) and 20 for other conditions and malformations (chest pain, paleness, double urethra).

Informed consent was obtained by the guardians of all children and the study protocol was approved by the Ethics Committee of the University of Athens Medical School. The parents of all cases and controls were interviewed in the hospital wards, in most instances by the same interviewer. The child's height and weight were abstracted from the medical records and transformed into age–gender-specific centiles, to allow comparisons across different ages, using growth curves developed for Greek children by the First Department of Pediatrics of the Athens Medical School (Chiotis *et al*, 2003). Information on the type of leukaemia was abstracted from the medical records and blood samples were collected during routine clinical procedures before the initiation of therapy.

All coded samples were centrifuged and frozen sera were sent in dry ice to the coordinating centre in Athens where they were stored at −70°C prior to being air shipped in one batch to the Beth Israel Deaconess Medical Center, in Boston, USA. Blinded adiponectin measurements were performed by a radioimmunoassay procedure with a sensitivity of 2 ng ml^−1^, and intra-assay coefficient of variation of <10% as previously described (Petridou *et al*, 2003; Dal Maso *et al*, 2004; Mantzoros *et al*, 2004b). Average preservation time was similar for cases and controls, and adiponectin levels do not systematically change with storage time.

Initially cases of childhood leukaemia, by type of disease, as well as control children were classified by gender, age and age–gender-specific centiles of height and weight. Representative values of adiponectin, by disease status, were subsequently calculated. In order to study a possible association of adiponectin with any of the three different types of childhood leukaemia, we modelled the data through multiple logistic regression in three distinct models using case/control status as the outcome variable and adiponectin (in increments of one s.d. of the hormone among controls) as well as gender, age and age–gender-specific centiles of height and weight as predictor variables. We have opted for unconditional logistic regression in order to utilise the adiponectin data from all variable controls in the crucial analysis concerning AML cases. Adjustment for covariates chosen as possible predictors of either adiponectin levels or disease risk (e.g. maternal smoking during pregnancy) and, thus, as conceivable confounders of the association of adiponectin levels and childhood leukaemia, have not altered the results appreciably (data not shown). The SAS statistical package was used in all analyses (SAS Institute Inc., 1989).

## RESULTS

Table 1 shows the distribution of 201 children with childhood leukaemia by disease type and 201 controls by gender, age and age–gender-specific centiles of height and weight. Since the gender and age matching was carried out for leukaemia cases overall differences in the distribution between controls and cases of the more rare forms of childhood leukaemia are evident. In the main analyses, however, gender and age were adjusted for, categorically, through unconditional logistic regression. Although there were no apparent differences with respect to height and weight, these factors were also adjusted for, as ordered variables, in the main analyses. In the multiple logistic regression analysis, age was also considered as continuous variable and no change was noted.

Table 2 shows representative values of adiponectin among children by different types of leukaemia and among control children. Adiponectin is approximately normally distributed and there is evidence in these bivariate analyses that levels of adiponectin are lower among children with AML than among apparently healthy controls. In contrast, there is no evidence for an important difference between control children on the one hand and children with ALL-B or ALL-T on the other.

In Table 3, we present multiple logistic regression-derived, adjusted odds ratios (OR) and 95% confidence intervals (CI) for different types of childhood leukaemia by adiponectin levels, controlling for gender, age as well as for height and weight in age–gender-specific centiles. There is no evidence that adiponectin is associated with either ALL-B or ALL-T. With respect to AML, however, adiponectin is inversely, significantly and strongly related to the disease. Weight is also significantly inversely associated with AML. Weight and height in age–gender-specific centiles are not strongly correlated among controls (Spearman's *r*=0.35) so that colinearity is not a major problem. Adjusting only for height or only for weight does not materially change the results. Thus, the OR for AML in relation to adiponectin, controlling for gender, age and for height only or weight only are: 0.61 (95% CI, 0.37–0.99) and 0.55 (95% CI, 0.34–0.92), respectively. Models, in which body weight and height were introduced as continuous variables (single centile increments), generated similar results (data not shown).

## DISCUSSION

In a nation-wide case–control study in Greece, we found evidence that adiponectin is inversely related to the risk of childhood AML, whereas no such evidence was found with respect to other forms of acute childhood leukaemia. These findings are in line with the working hypothesis generated by a previous report that adiponectin, a hormone that induces apoptosis, inhibits proliferation of myeloid, but not of lymphoid, cell lines (Yokota *et al*, 2000).

Adiponectin, also called gelatin-binding protein-28, is a 244 amino-acid protein secreted exclusively from white adipose tissue, mainly visceral adipose tissue, to the amount of which it is inversely related (Motoshima *et al*, 2002). Adiponectin acts principally as an insulin sensitiser; it is inversely associated with parameters of adult obesity and has anti-inflammatory and antiatherogenic effects (Arita *et al*, 1999; Ouchi *et al*, 1999; Yokota *et al*, 2000; Ouchi *et al*, 2001; Motoshima *et al*, 2002; Cnop *et al*, 2003; Gavrila *et al*, 2003). Four epidemiological studies on adiponectin in relation to cancer have been undertaken among adults and all four have pointed to inverse associations of the hormone with breast cancer (Miyoshi *et al* 2003; Mantzoros *et al* 2004b) and endometrial cancer (Petridou *et al*, 2003; Dal Maso *et al*, 2004), notably with the postmenopausal forms of the two diseases. Importantly, adiponectin has been reported to induce apoptosis through activation of members of the caspase group of apoptotic enzymes (Brakenhielm *et al*, 2004) including apoptosis in myelomonocytic leukaemia lines and to predominantly inhibit proliferation of myeloid but not of erythroid or lymphoid cell lines. Treatment of acute myelomonocytic leukaemia lines with adiponectin induces the appearance of subdiploid peaks and oligonucleosomal DNA fragmentation. Moreover, adiponectin regulates inflammatory responses negatively through at least two mechanisms: inhibition of growth of macrophage precursors and suppression of mature macrophage functions (Yokota *et al*, 2000).

Exclusion of cases and controls from a particular catchments area restricts the study base but should not, by itself, compromise validity. Case ascertainment in our investigation was population based and exclusions were based on administrative or technical reasons that are unlikely to have introduced selection bias. Since blood samples were necessary, we opted for inclusion of hospital controls, which were carefully matched to cases, whereas their admission diagnoses are not known to be related with the principal exposure variable, namely adiponectin. Consequently, there were very few refusals for cooperation among the guardians of both the cases and controls. Adiponectin measurements were blindly performed in a certified laboratory and none of the diagnoses of control subjects have been associated with increased adiponectin levels.

We did not control for components of the insulin-like growth factor (IGF) system; the limited existing evidence for this system refers to ALL rather than AML (Ross *et al*, 1996; Petridou *et al*, 2000) and the association between adiponectin and IGF-1 in children is not strong enough to generate considerable confounding (Mantzoros *et al*, 2004a). Control for height, which is positively and weight, which is inversely associated with adiponectin levels (Mantzoros *et al*, 2004a), was carried out by using the corresponding age–gender-specific centiles in growth curves among Greek children (Chiotis *et al*, 2003), thus allowing fine adjustment despite the variable ages of cases and controls ranging between 0 and 14 years of age. We have not controlled for birth weight, both because the evidence for it being associated with childhood leukaemia risk refers mostly to ALL (Hjalgrim *et al*, 2003, 2004), and to avoid in the small data set of AML, the consequences of the ‘reversal paradox’ phenomenon generated when body weight, which is related to birth weight, is found to be a strong predictor of the disease, as was the case in our study (Tu *et al*, 2005). Children with leukaemia and control children were generally taller and heavier than predicted from the growth curves for Greek children (Chiotis *et al*, 2003) but this does not affect the ability to control confounding through use of the corresponding age–gender-specific centiles in these scales. MacInnis *et al* (2005) reviewed among other issues that risk of myeloid leukaemia among adults 27–75 years old, was positively associated with body mass index whereas lymphoproliferative malignancies and subgroups showed little relationship with body size. We have not been able to document in this data set this association of weight and childhood leukaemia in the univariate analysis. It remains to be investigated whether these divergences are imposed by the small size of our study or differences in the biology between children and older adults. A possible explanation could be that adult height, BMI etc. reflect the life-long effect of the IGF system which also increases risk for leukaemia whereas in children the exposure to the IGF system is not long enough to show positive associations. The strong inverse association of childhood AML with weight that was found in the multivariate analysis of our study has not been previously documented in childhood. Irrespective of whether it is genuine or not, it should not confound the association of adiponectin with AML, given that it is appropriately controlled for.

In conclusion, we have found biologically plausible and empirically strong evidence that among children, adiponectin levels are inversely associated with AML, in contrast to other types of childhood leukaemia. Although adiponectin has generally been considered as a hormone-modulating insulin resistance-related phenomenon in adult life (Arita *et al* 1999; Stefan *et al*, 2002; Cnop *et al*, 2003; Gavrila *et al*, 2003) the very high levels of this hormone in early life (Mantzoros *et al*, 2004a) suggest an important role of adiponectin during this period as well. Our *in vivo* data in combination to the experimental findings by Yokota *et al* suggest that adiponectin may have a specific role in leukaemia which needs to be explored further in the future. Since there were only 22 children with AML in our study, an unavoidable consequence of the rarity of the disease and the requirement for obtaining blood samples from very young children, our results cannot be considered as conclusive. If confirmed, however, they may provide a unique insight on the pathogenesis of childhood AML and on the early life physiological and pathophysiological role of adiponectin.

## Figures and Tables

**Table 1:** Distribution of 201 children with leukaemia by type and 201 control children by gender age and age–gender-specific centiles of height and weight

	**ALL-B**		**ALL-T**		**AML**		**Controls**	
**Variable**	***N***	**%**	***N***	**%**	***N***	**%**	***N***	**%**
*Gender*								
Male	77	47.8	13	72.2	10	45.5	100	49.8
Female	84	52.2	5	27.8	12	54.5	101	50.2

*P-value (vs controls; χ*^*2*^ *1 df)*	0.71		0.07		0.70			

*Age (years)*								
<1	2	1.2	0	0.0	3	13.6	4	2.0
1–4	90	55.9	6	33.3	12	54.6	108	53.7
5–9	48	29.8	8	44.5	2	9.1	60	29.9
10+	21	13.1	4	22.2	5	22.7	29	14.4

*P-value for trend (vs controls; χ*^*2*^ *3 df)*	0.92		0.32		0.01			

*Height (age–gender-specific centiles)*								
<3	5	3.1	0	0.0	1	4.6	5	2.5
3–24	23	14.3	3	16.7	2	9.1	26	12.9
25–49	24	14.9	3	16.7	0	0.0	30	14.9
50–74	34	21.1	3	16.7	6	27.2	37	18.4
75–96	54	33.5	7	38.8	10	45.5	70	34.9
97+	20	13.1	2	11.1	3	13.6	33	16.4

*P-value for trend (vs controls; χ*^*2*^ *1 df)*	0.38		0.80		0.48			

*Weight (age–gender-specific centiles)*								
<3	2	1.3	1	5.6	1	4.6	5	2.5
3–24	25	15.5	5	27.8	6	27.2	26	12.9
25–49	38	23.6	2	11.1	5	22.7	37	18.4
50–74	42	26.1	3	16.6	4	18.2	48	23.9
75–96	44	27.3	6	33.3	4	18.2	66	32.8
97+	10	6.2	1	5.6	2	9.1	19	9.5

*P-value for trend (vs controls; χ*^*2*^ *1 df)*	0.16		0.22		0.06			

**Table 2:** Representative values of adiponectin by type of leukaemia and controls

**Adiponectin (*μ*g ml^−1^)**	**ALL-B**	**ALL-T**	**AML**	**Controls**
Number	161	18	22	201
Median	17.7	16.8	14.8	19.2
Mean	18.6	19.4	15.8	19.4
s.d.	7.8	10.6	7.4	7.9
*P-value*	*0.33*	*0.96*	*0.048*	

*P*-value derived from *t*-tests, comparing means between leukaemia types and controls.

**Table 3:** Multiple logistic regression-derived, adjusted odds ratios (OR) and 95% confidence intervals (95% CI) for different types of childhood leukaemia by anthropometric variables and adiponectin levels

**Variable**	**Category or increment**	**OR**	**95% CI**		***P*-value**
*Model 1*: *ALL-B cases and 201 controls*					
Gender					
	Male	Baseline			
	Female	1.11	0.73	1.69	0.62
Age (years)					
	<5	Baseline			
	5–9	0.88	0.54	1.43	0.60
	10–14	0.75	0.39	1.44	0.39
Height centile^a^					
	1 category more	0.97	0.82	1.15	0.71
Weight centile^a^					
	1 category more	0.88	0.73	1.06	0.19
Adiponectin					
	1 s.d. (among controls)	0.88	0.71	1.10	0.26

*Model 2: ALL-T cases and 201 controls*					
Gender					
	Male	Baseline			
	Female	0.40	0.13	1.16	0.09
Age (years)					
	<5	Baseline			
	5–9	2.17	0.69	6.76	0.18
	10–14	2.50	0.63	9.89	0.19
Height centile^a^					
	1 category more	1.10	0.73	1.66	0.64
Weight centile^a^					
	1 category more	0.82	0.54	1.24	0.34
Adiponectin					
	1 s.d. (among controls)	1.08	0.67	1.72	0.76

*Model 3: AML cases and 201 controls*					
Gender					
	Male	Baseline			
	Female	1.21	0.48	3.07	0.68
Age (years)					
	<5	Baseline			
	5–9	0.18	0.04	0.84	0.03
	10–14	0.87	0.26	2.91	0.81
Height centile^a^					
	1 category more	1.32	0.88	1.98	0.18
Weight centile^a^					
	1 category more	0.61	0.41	0.89	0.01
Adiponectin					
	1 s.d. (among controls)	0.56	0.34	0.94	0.03

a Age- and gender-specific centiles.

## Appendix A

The Childhood Hematology-Oncology Group: **M Moschovi**, Hematology-Oncology Unit, First Department of Pediatrics, Athens University Medical School, ‘Aghia Sophia’ General Children's Hospital, Athens, Greece. **F Athanassiadou- Piperopoulou**, 2nd Department of Pediatrics, Aristotle University of Thessaloniki, American Hellenic Educational Progressive Association General Hospital, Thessaloniki, Greece. **S Polychronopoulou**, Department of Pediatric Hematology-Oncology, ‘Aghia Sophia’ General Children's Hospital, Athens, Greece. **M Baka**, Department of Pediatric Hematology-Oncology, ‘Pan.&Agl. Kyriakou’ Children's Hospital, Athens, Greece. **M Kalmanti**, Department of Pediatric Hematology-Oncology, University Hospital of Heraklion, Heraklion, Greece.
